# Beyond Gut Instinct: Metabolic Short-Chain Fatty Acids Moderate the Pathogenesis of Alphaherpesviruses

**DOI:** 10.3389/fmicb.2019.00723

**Published:** 2019-04-05

**Authors:** Katrien C. K. Poelaert, Jolien Van Cleemput, Kathlyn Laval, Sarah Descamps, Herman W. Favoreel, Hans J. Nauwynck

**Affiliations:** ^1^Laboratory of Virology, Department of Virology, Immunology and Parasitology, Faculty of Veterinary Medicine, Ghent University, Merelbeke, Belgium; ^2^301 Schultz Laboratory, Department of Molecular Biology, Princeton University, Princeton, NJ, United States

**Keywords:** alphaherpesviruses, varicellovirus, short-chain fatty acids, pathogenesis, respiratory tract, viremia, endothelium

## Abstract

Short-chain fatty acids (SCFA), such as sodium butyrate (SB), sodium propionate (SPr), and sodium acetate (SAc), are metabolic end-products of the fermentation of dietary fibers. They are linked with multiple beneficial effects on the general mammalian health, based on the sophisticated interplay with the host immune response. Equine herpesvirus 1 (EHV1) is a major pathogen, which primarily replicates in the respiratory epithelium, and disseminates through the body via a cell-associated viremia in leukocytes, even in the presence of neutralizing antibodies. Infected monocytic CD172a^+^ cells and T-lymphocytes transmit EHV1 to the endothelium of the endometrium or central nervous system (CNS), causing reproductive or neurological disorders. Here, we questioned whether SCFA have a potential role in shaping the pathogenesis of EHV1 during the primary replication in the URT, during the cell-associated viremia, or at the level of the endothelium of the pregnant uterus and/or CNS. First, we demonstrated the expression of SCFA receptors, FFA2 and FFA3, within the epithelium of the equine respiratory tract, at the cell surface of immune cells, and equine endothelium. Subsequently, EHV1 replication was evaluated in the URT, in the presence or absence of SB, SPr, or SAc. In general, we demonstrated that SCFA do not affect the number of viral plaques or virus titer upon primary viral replication. Only SB and SPr were able to reduce the plaque latitudes. Similarly, pretreatment of monocytic CD172a^+^ cells and T-lymphocytes with different concentrations of SCFA did not alter the number of infected cells. When endothelial cells were treated with SB, SPr, or SAc, prior to the co-cultivation with EHV1-inoculated mononuclear cells, we observed a reduced number of adherent immune cells to the target endothelium. This was associated with a downregulation of endothelial adhesion molecules ICAM-1 and VCAM-1 in the presence of SCFA, which ultimately lead to a significant reduction of the EHV1 endothelial plaques. These results indicate that physiological concentrations of SCFA may affect the pathogenesis of EHV1, mainly at the target endothelium, in favor of the fitness of the horse. Our findings may have significant implications to develop innovative therapies, to prevent the devastating clinical outcome of EHV1 infections.

## Introduction

In the last decades it became clear that nutrition has a large impact on the immune system of all mammals. Chronic overnutrition with high-fat meals or rapidly digestible carbohydrates leads to accumulation of fat in the adipose tissue, which subsequently becomes infiltrated with immune cells, inherent with increased systemic concentrations of inflammatory mediators, such as tumor necrosis factor alpha (TNFα) and interleukin-6 (IL-6) (Kolb and Mandrup-Poulsen, 2010; Morrison and Preston, 2016). In contrast, increased consumption of dietary fibers is associated with the reduction of systemic inflammation and immune disorders (Esposito and Giugliano, 2006; Anderson et al., 2009). The metabolites of dietary fibers, such as short chain fatty acids (SCFA) could explain these anti-inflammatory effects. SCFA, such as butyrate (C4), propionate (C3) and acetate (C2), are fermentation metabolites of carbohydrates produced by the intestinal microbiome (den Besten et al., 2013). In the mammalian gastro-intestinal tract, SCFA are present in high concentrations in the lumen of the large intestine, where they are actively metabolized by the colonic epithelium to produce energy. Moreover, SCFA are absorbed by the colonic epithelial cells, pass the liver, and consequently enter the systemic circulation in low, but measurable concentrations (0.1–10 mM) (Comalada et al., 2006; Pluznick, 2014). SCFA have been linked with beneficial effects in the gastro-intestinal inflammatory disorders and protection from colon cancer in humans. However, little information is available on the consequences of dietary fibers on local and/or systemic inflammation and infections in the mammalian body, including at the respiratory tract and endothelium. SCFA are thought to elicit their effects via multiple ways. Firstly, SCFA bind to endogenous G-protein coupled receptors FFA2 and FFA3. Both receptors are widely expressed on cells, including immune cells, adipocytes, endothelial cells, and respiratory epithelium (Li et al., 2018). Binding of SCFA to FFA2, or FFA3 leads to engagement of the multifunctional adaptor protein, β-arrestin, triggering the MAPK signaling, which ultimately culminates in the activation of transcription factors, important for cell growth, proliferation, and survival (Smith and Rajagopal, 2016). Butyrate is one of the best-studied SCFA, known to decrease several pro-inflammatory cytokines, such as TNFα and IL-12, and to upregulate production of the anti-inflammatory cytokine IL-10. Moreover, butyrate is associated with the phosphorylation of STAT-1 and STAT-2, one of the first steps in the IFN-signaling pathway (Nusinzon and Horvath, 2003; Dedoni et al., 2016). Secondly, mainly butyrate, and to a lesser extent propionate inhibit histone deacetylase activity. Histone deacetylases (HDAC) are ubiquitously expressed in the nucleus and cytoplasm of immune cells, endothelial cells, and vascular muscle cells (Gray and Ekstrom, 2001; Zhou et al., 2011; Didonna and Opal, 2015). Inhibiting nuclear HDAC activity results in the enhanced acetylation of histone proteins, ultimately leading to an open structure of the chromatin, and enhanced gene transcription (Reichert et al., 2012).

Horses are experts in hindgut fermentation and are sensitive to small dietary changes, making them an ideal model to analyze the consequences of dietary variation on mammal health (Argenzio, 1975; Janis, 1976; Burkhardt et al., 1997). The main colonizers within the colon and caecum of *Equidae* are proteolytic bacteria, such as *Streptococcus* spp., and *Bacteroides* spp., lactate-utilizing bacteria, predominantly *Megasphaera* spp. and *Veillonella* spp., cellulolytic and fibrolytic bacteria, such as *Butyrivibrio* spp., *Clostridium* spp., *Eubacterium* spp., and *Ruminococcus* spp. (Daly et al., 2001; Julliand, 2005; Dicks et al., 2014). It is known that a nutrition-related disbalance between those micro-organisms can lead to a decreased pH, which ultimately might result in lactate acidosis, colic, anorexia and in predisposing animals to bouts of laminitis (Biddle et al., 2013). Moreover, alterations in the intestinal micro-environment have also been correlated with changes in the course of several human respiratory diseases, including asthma (De Filippo et al., 2010; Bisgaard et al., 2011; Abrahamsson et al., 2014; Bruzzese et al., 2014). However, whether these dietary factors also affect responses against respiratory and systemic viral infections is still unknown. In this study, we addressed the role of the dietary metabolites butyrate, propionate and acetate on the pathogenesis of one of the most important equine alphaherpesvirus, the ancient equine herpesvirus 1 (EHV1) (Karlin et al., 1994).

Horses usually become infected with EHV1 within the first year of life, which cannot be prohibited by current vaccines (Lunn et al., 2009). The virus can spread via respiratory secretions during (in)direct contact between horses. Upon infection, EHV1 replicates in the epithelium of the upper respiratory tract (URT), crosses the basement membrane and enters the blood circulation in single infected immune cells (Gryspeerdt et al., 2010; Vandekerckhove et al., 2010). EHV1 has evolved the ability to evade the immune surveillance, e.g., by misusing monocytic CD172a^+^ cells and T-lymphocytes as transport vehicles to reach the endothelium of the pregnant uterus, or central nervous system (CNS). Infection of the target endothelium often results in ischemia and thrombo-embolic disease, eventually causing neonatal foal death, late-term abortion or myelo-encepthalopathy (EHM) (Edington et al., 1986, 1991; van der Meulen et al., 2000; Goehring et al., 2006; Laval et al., 2015a). So far, little information is known about the role of nutritional metabolites on the pathogenesis of EHV1. Only one study of Laval et al. (2015a) demonstrated that the replication of the abortigenic EHV1 strains in monocytic cells is silenced by HDAC at the level of the viral gene transcription. Treatment of infected mononuclear cells with butyrate, which suppress HDAC activity, resulted in the activation of the viral replication. However, the consequences of SCFA during primary viral replication, infection of immune cells, and viral transfer to the target endothelium remains unclear. We hypothesized that SCFA may impede EHV1 infection of the URT, by hindering virus entry and/or viral spread in the respiratory epithelium. Secondly, we hypothesized that SCFA may change the phenotype of monocytic CD172a^+^ cells and T-lymphocytes, the main target cells of EHV1, affecting their susceptibility to viral infection. Thirdly, since SCFA are known for their anti-inflammatory properties, we theorized that SCFA may prevent viral transfer from infected mononuclear cells to the engaged endothelial cells. Understanding the fragile balance between host immunity, metabolic factors, and the viral pathogenesis may be of key importance to prevent and/or cure the sometimes devastating consequences of alphaherpesvirus infections.

## Materials and Methods

### Virus

One Belgian EHV1 strain was included in this study and was genotyped in the ORF30 region by the Animal Health Trust in the United Kingdom. The 97P70 EHV1 strain, encoding an asparagine at amino acid position 752 (N_752_) (Nugent et al., 2006), was originally isolated in 1997 from the lungs of an aborted fetus (van der Meulen et al., 2003; Van de Walle et al., 2009). Virus stock of 97P70 was used for inoculation at the 6th passage. The last passage was performed in RK-13 cells.

### Respiratory Mucosa Explants and Cells

#### Respiratory Mucosa Explants

Respiratory tissues from horses were collected *post mortem* in the slaughterhouse, approved by the Ethical committee of Ghent University (2018_NOPROC_01). Horses negative for ocular/nasal discharge and lung pathologies were selected. The horses were aged between 5 and 15 years old, as determined by inspection of dental incisive architecture (Muylle et al., 1996). Each experiment was conducted with tissues from three different horses. The proximal part of the trachea was collected from each horse. Tissues were transported to the laboratory on ice, in phosphate-buffered saline (PBS), supplemented with 1% gentamycin, 1% penicillin-streptomycin (Gibco, Invitrogen, Paisley, United Kingdom), 1% kanamycin (Sigma-Aldrich, St. Louis, MO, United States) and 0.5% amphotericin B (Bristol-Myers Squibb). Respiratory mucosa explants were collected and cultivated as described previously by Vandekerckhove et al. (2010) and Poelaert et al. (2018).

#### Cells

##### Isolation of equine blood CD172a^+^ cells and T-lymphocytes

Peripheral blood was sampled from the external *vena jugularis* into heparin (Leo, Zaventem, Belgium) at a final concentration of 15 U ml^-1^. The Ethical Committee of the Faculty of Veterinary Medicine, Ghent University (application EC2017/118) approved the collection of blood. Isolation of PBMC was conducted as previously described by Poelaert et al. (2019). Briefly, fresh blood was diluted in an equal volume of Dulbecco’s phosphate-buffered saline (DPBS) without calcium and magnesium (Gibco). Peripheral blood mononuclear cells (PBMC) were isolated by density centrifugation on Ficoll-Paque (*d* = 1.077 g ml^-1^) (GE Healthcare, Life Sciences) at 800 × *g* for 30 min at 18°C. The interphase band, containing the PBMC, was collected and washed three times with DPBS. Cells were resuspended in complete medium based on Roswell Park Memorial Institute (RPMI) (Gibco), supplemented with 5% newborn fetal calf serum (FCS) (Gibco), 1% non-essential amino acids, 1% sodium pyruvate, 1% penicillin-streptomycin, 0.5% gentamycin (Gibco). Afterward, PBMC were seeded at a concentration of 5 × 10^6^ cells per ml and cultivated at 37°C with 5% CO_2_. After 2 h, non-adhering leukocytes were removed by washing cells three times with RPMI and the adherent cells were cultured in complete medium. Non-adherent cells were washed 3 times with ice-cold RPMI. The cell pellet was incubated with 400 μl of mouse monoclonal (mAb) anti-horse pan B-cell, clone CVS36 (IgG_1_; 1:50) (Biorad, United States) diluted in ice-cold DPBS, for 1 h at 4°C with gentle agitation. Afterward, cells were washed once in magnetic-activated cell sorting (MACS) buffer containing DPBS supplemented with 1 mM EDTA and 5% newborn FCS and incubated with 400 μl rat anti-mouse IgG_1_ microbeads (MACS Miltenyi Biotec, Bergisch Gladbach, Germany) diluted in MACS buffer (1:5), for 1 h at 4°C with gentle agitation. Next, the cells were washed once in MACS buffer and resuspended in 3 ml MACS buffer for application to the MACS column. The unbound cells of the pan-B-cell antibody-incubated T-lymphocytes were collected from the column and consisted of >90% of CD3^+^ cells, assessed by flow cytometry after incubation with a mAb anti-CD3, clone UC-F6G (IgG_1_; 1:50) (UC Davis, Davis, CA, United States), directed against cells from the T-cell lineage, followed by goat anti-mouse IgG FITC (1:200) (Molecular Probes) (data not shown). All T-lymphocytes were cultured in complete medium supplemented with 4 U ml^-1^ human recombinant interleukin-2 (hIL-2) (R&D systems) and 50 mM β-mercaptoethanol (β-ME) (Gibco).

##### Equine venous endothelial cell culture

Laval et al. (2015b) previously reported the isolation and immortalization of equine venous endothelial cells (EC). EC were maintained in EC medium containing Dulbecco’s modified Eagle Medium (DMEM) (Gibco) supplemented with 5% FCS and antibiotics. Firstly, to analyze the effects of Sodium Butyrate (SB), Sodium Propionate (SPr), or Sodium Acetate (SAc) (Sigma-Aldrich) on the expression of adhesion molecules on the EC plasma membrane, EC were grown to confluence in 6-well plates coated with 10 μg ml^-1^ fibronectin from bovine plasma (Sigma-Aldrich). Twenty-four hours prior to collection, EC were cultured in EC medium supplemented with 0 or 5 mM SCFA. Previous studies demonstrated that lipopolysaccharide (LPS) stimulation of vascular endothelium increases the expression of ICAM-1 and VCAM-1 on the cellular plasma membrane (Faure et al., 2000; Hijiya et al., 2002; Zeuke et al., 2002; Dauphinee and Karsan, 2006). Therefore, in this study EC were pretreated 3 h prior to collection with 0 or 10 μg ml^-1^ LPS derived from *Escherichia coli* (Sigma-Aldrich), to analyze the effects of SCFA on the expression of the adhesion molecules.

Secondly, to analyze the co-cultivation of EHV1-inoculated mononuclear cells and EC in the presence or absence of SCFA, EC were grown to confluence in 8-well Lab-Tek II chamber slides (Thermo Fisher Scientific, Rochester, NY, United States) coated with 10 μg ml^-1^ fibronectin. Six hours prior to co-cultivation with PBMCs, medium was substituted by EC medium supplemented with 0, 0.5, or 5 mM SB, SPr, or SAc.

Finally, EC were cultured as previously described in 8-well Lab-Tek II chamber slides and treated with 0.5 or 5 mM SB, SPr, or SAc during 24 h prior to EHV1 inoculation. Different concentrations of SCFA were maintained throughout inoculation and cultivation of EC.

##### Rabbit kidney epithelial (RK13) cells

Rabbit kidney epithelial cells were purchased from the American Type Culture Collection (ATCC, Manassas, VA, United States) and were used in this study to analyze EHV1 replication, by quantifying extracellular virus titers at different time points post inoculation. Cells were maintained in MEM supplemented with antibiotics and 5% FCS.

### Cell Viability

Cell viability of the SCFA-pretreated mononuclear cells and EC was determined by flow cytometry, using 1 μg ml^-1^ propidium iodide (Sigma-Aldrich), prior to virus inoculation and was >90% in all cell populations. An *in situ* Cell Death Detection Kit (Fluorescein) based on Terminal deoxynucleotidyl transferase mediated dUTP Nick End Labeling (TUNEL) was obtained from Roche (Mannheim, Germany) and used to detect DNA fragmentation induced by apoptotic signaling cascades. The co-localization of incorporated dUTP in respiratory mucosa epithelium and lamina propria was analyzed with fluorescence microscopy (Leica DM RBE microscope, Leica Microsystems GmbH, Heidelberg, Germany). The number of TUNEL positive cells was evaluated in five randomly chosen fields of the epithelium and the *lamina propria*. No significant differences were observed between SCFA-treated mucosa explants and the control (data not shown).

### EHV1 Inoculation

#### Pretreatment and Inoculation of Respiratory Mucosa Explants

Serum-free medium (DMEM/RPMI, supplemented with antibiotics) containing 0, 0.5 or 5 mM SB, SPr or SAc, were added 24 h prior to inoculation and maintained throughout the inoculation and cultivation of the explants. After 24 h of culture, explants were taken from their gauzes and placed in a 24-well plate with the epithelial surface upward. Warm serum-free medium was used to wash them twice. Explants were inoculated with EHV1, as previously described by Vandekerckhove et al. (2010). Briefly, inoculation took place by the immersion of the explant in 1 ml inoculum, containing 10^6.5^ TCID_50_ of EHV1, for 1 h at 37°C. After inoculation, explants were washed twice in warm serum-free medium and transferred to their gauzes. At 24 hpi mucosa explants were collected and embedded in methylcellulose medium (Methocel^®^ MC, Sigma-Aldrich) and frozen at -70°C. Supernatant was collected and stored at -70°C until further use.

#### Pretreatment and Inoculation of Monocytic CD172a^+^ Cells and T-Lymphocytes

CD172a^+^ monocytic cells and T-lymphocytes were inoculated with EHV1 at a multiplicity of infection (MOI) of 5, for 1 h at 37°C. Where mentioned, complete medium supplemented with 0, 0.5 or 5 mM SB, SPr or SAc was added 24 h prior to inoculation and was maintained throughout the (mock)-inoculation and cultivation of the mononuclear cells.

#### Binding and Co-cultivation Assay of EHV1-Inoculated Mononuclear Cells With Equine Endothelial Cells

To analyze the PBMC binding and EHV1 transfer to equine EC in the presence of SCFA, we assessed a PBMC binding assay and co-cultivation assay. Briefly, mock- or EHV1 inoculated monocytic CD172a^+^ cells or T-lymphocytes were collected at 12 hpi and washed with citrate buffer containing 40 mM citric acid, 135 mM NaCl, 10 mM KCl (pH 3), during 1 min at RT, to neutralize cell-free virus particles. Cells were rinsed three times, to remove redundant citrate buffer, and T-lymphocytes were resuspended in 200 μl complete medium supplemented with 0, 0.5 or 5 mM SB, SPr or SAc. CD172a^+^ monocytic cells were detached by incubating with pure Accumax^TM^ solution (Sigma-Aldrich) for 30 min at 37°C, and were collected in ice-cold falcon tubes containing FCS to neutralize the enzyme activity. Monocytic cells were resuspended in 200 μl ice-cold complete medium supplemented with 0, 0.5 or 5 mM SB, SPr or SAc. Both subsets of mononuclear cells were layered on SCFA-treated equine EC monolayers, prior to co-cultivation (ratio of 2:1). Cells were further co-cultured for 15 min, 60 min (binding assay) or 36 h (co-cultivation assay) at 37°C, 5% CO_2_. EC and adherent mononuclear cells were fixed with 100% methanol (-20°C, 20 min) and stored in -20°C until further processing.

#### Pretreatment and Inoculation of Equine Venous Endothelial Cells

Endothelial cells medium (DMEM, supplemented with 5% FCS and antibiotics) containing 0, 0.5 or 5 mM SB, SPr or SAc, was added 24 h prior to inoculation and maintained throughout the inoculation and cultivation of the EC. After 24 h of culture, EC were washed twice in DMEM containing different concentrations of SCFA. EC were inoculated at a MOI of 5, for 1 h at 37°C. At 24 hpi, EC were fixed and supernatants was collected.

### Virus Titrations

The extracellular viral titer was determined in supernatant from respiratory mucosa explants, monocytic CD172a^+^ cells, T-lymphocytes, and EC inoculated with cell-free virus. Virus titers were assessed by a 50% tissue culture infective dose assay using RK13 cells. The 50% end-point was calculated according to the method of Reed and Muench (1938).

### Bioassay for Determining Interferon Antiviral Activity

Supernatants of EHV1- and mock-inoculated SCFA-pretreated tracheal explants were harvested at 24 hpi. Type I interferon (IFN) bioactivity was determined by a cytopathic effect (CPE) reduction assay based on vesicular stomatitis virus (VSV) and Madin-Darby bovine kidney (MDBK) cells as previously described in La Bonnardiere and Laude (1981) and Poelaert et al. (2018). Briefly, MDBK cells were seeded in 96-well micro titer plates in DMEM with 5% FCS, 1% sodium pyruvate, 1% penicillin-streptomycin solution and 1% gentamycin. After overnight incubation at 37°C, 5% CO_2_, medium was removed and serial two-fold dilutions of samples were added to the confluent cells. Following 18 h incubation (37°C, 5% CO_2_), 50 μl of VSV was added to the samples and virus control wells, at a concentration resulting in complete CPE after 48 h. To the cell control wells, only 50 μl of medium was added. Following 48 h incubation, medium was aspirated and 50 μl of 0.1% neutral red solution was added to the cells during 1 h at 37°C in 5% CO_2_. Next, cells were rinsed, air-dried and 150 μl of dissolving solution (50 μl sodium lauryl sulfate (SDS), 100 μl 0.2 M HCl in H_2_O) was added. The absorbance of neutral red solution at 492 nm was determined with a micro-plate reader. The IFN titer was calculated as the reciprocal of the last IFN dilution causing 50% inhibition of virus-induced CPE and was expressed as IFN units per volume. Recombinant EqIFNα (Kingfisher Biotech Inc., Saint Paul, MN, United States) with a titer of 18 × 10^3^ U ml^-1^ was run in each assay as positive control. The relative IFN (rIFN) bioactivity upon SCFA treatment was calculated by standardizing the values with the correspondent extracellular virus titer.

### Indirect Immunofluorescence Staining

#### SCFA Receptors

Fifty consecutive cryosections of 16 μm were made of the respiratory and lymph nodal tissues derived from three individual horses. The frozen sections were mounted on 3-aminopropyltriethoxysilane (Sigma-Aldrich) coated slides, and fixed in 4% paraformaldehyde (PFA) for 10 min at room temperature (RT). The sections were washed two times in DPBS, and permeabilized in 0.1% Triton X-100, during 10 min at RT. Subsequently, the sections were rinsed twice in DPBS, and incubated with a rabbit polyclonal antibody (pAb) against GPR43 (IgG, 1:50 in DPBS) or rabbit pAb against GPR41 (IgG, 1:50 in DPBS) (Thermo Fisher Scientific) to visualize FFA2 or FFA3, respectively. Subsequently, the sections were incubated with a FITC-conjugated goat anti-rabbit IgG antibody (1:100 in DPBS) (Molecular Probes, Eugene, OR, United States). All antibodies were incubated for 1 h at 37°C. Cell nuclei were counterstained with Hoechst 33342 (10 μg ml^-1^) (Molecular probes).

#### Viral Proteins

##### Respiratory mucosa explants

At 24 hpi, fifty consecutive cryosections of 16 μm were made of the frozen explants derived from three horses. The frozen sections were mounted on 3-aminopropyltriethoxysilane coated slides. They were fixed in 100% methanol for 20 min at -20°C, and then washed with DPBS. Late viral proteins were stained with biotinylated equine polyclonal anti-EHV1 IgG antibody (1:20 in DPBS) (van der Meulen et al., 2003), followed by streptavidin FITC (1:200 in DPBS) (Molecular Probes). Subsequently, the basement membrane of the explants was stained by incubation with a mouse monoclonal anti-collagen VII IgG1 antibody (clone LH7.2; 1:50 in DPBS) (Sigma-Aldrich), followed by a Texas Red^®^-conjugated goat anti-mouse IgG antibody (1:200 in DPBS) (Molecular Probes). Antibodies were incubated for 1 h at 37°C. Cell nuclei were counterstained with Hoechst 33342 (10 μg ml^-1^). Determinations of viral plaque numbers and plaque latitude were data obtained from 50 consecutive cryosections.

##### Mononuclear cells

Monocytic CD172a^+^ cells and T-lymphocytes were collected at 12 hpi. Monocytic CD172a^+^ cells were fixed in 100% methanol for 20 min at -20°C, whereas T-lymphocytes were fixed in 1% PFA during 10 min at RT and washed two times with DPBS. T-lymphocytes were prepared for immunofluorescence staining using cytospin-centrifugation (7 min, 700 rpm). Next, T-cells were permeabilized with 0.1% Triton X-100 during 2 min at RT. All mononuclear cells were incubated for 1 h at 37°C with a rabbit pAb against IEP (1:1000) (Smith et al., 1994; Jang et al., 2001) to visualize immediate early protein (IEP). The IEP antibody was kindly provided by Prof. Dr. O’Callaghan (United States). Subsequently, cells were incubated during 50 min at 37°C with goat anti-rabbit IgG FITC (1:100). All antibodies were diluted in DPBS. Cell nuclei were counterstained with Hoechst 33342 (10 μg ml^-1^) (Molecular probes).

##### Equine venous endothelial cells

Firstly, to visualize the transfer of infection from EHV1-inoculated mononuclear cells to EC a double immunofluorescence staining was carried out after 36 h of co-cultivation. Late viral proteins were stained with the biotinylated pAb against EHV1 (1:20). Next, cells were incubated during 50 min at 37°C with streptavidin FITC (1:200) and nuclei were counterstained with Hoechst 33342 (10 μg ml^-1^). Secondly, to visualize the viral replication in EC in presence of different concentrations of SCFA, cells were incubated during 1 h at 37°C with the biotinylated pAb against EHV1 (1:20), to visualize late viral proteins. Next, EC were incubated with streptavidin FITC (1:200) during 50 min, 37°C. Cell nuclei were counterstained with Hoechst 33342 (10 μg ml^-1^).

##### Adhesion molecules

To analyze the effects of SCFA on equine EC, an indirect immunofluorescence staining for ICAM-1 and VCAM-1 was carried out. Briefly, EC pretreated with 0 or 5 mM SB, SPr, or SAc were detached with pure Accumax^TM^ solution for 10 min, 37°C, and were collected in ice-cold falcon tubes containing FCS to neutralize the enzyme activity. Cells were immediately fixed in 1% PFA for 10 min at RT, rinsed twice with DPBS, followed by cytospin centrifugation. EC were washed and incubated with a mouse monoclonal antibody (mAb) against ICAM-1 (IgG_1_, 1:50 in DPBS) (Invitrogen), mouse mAb against VCAM-1 (IgG_2a_, 1:50 in DPBS) (Biorbyt Ltd., Cambridge, United Kingdom) or irrelevant isotype controls during 60 min at 4°C. Next, cells were incubated during 60 min at 4°C with goat anti-mouse IgG TR (1:100 in DPBS). Cell nuclei were counterstained with Hoechst 33342 (10 μg ml^-1^).

### Confocal Microscopy

Immunofluorescence staining of all cells was analyzed by confocal microscopy (Leica TCS SP2 Laser Scanning Spectral Confocal System; Leica Microsystems). A Gre-Ne 543 nm laser was used to excite Texas Red-fluorochromes. An Argon 488 nm laser excited FITC-fluorochromes.

### Statistical Analysis

Analyzed data for statistical significance were subjected to a multiple-way analysis of variance (ANOVA). The Dunnett-test was used as a *post hoc* test. If the assumption of equal variables was not fulfilled with the Levene’s test, the data were log-transformed prior to ANOVA. Normality of the residuals was verified by the use of the Shapiro–Wilk test. A Kruskall–Wallis’ test, followed by a Mann–Whitney’s *post hoc* test was performed when variables remained unequal or when normality was not achieved after log-transformation. Differences in results with *p*-values < 0.05 were considered statistically significant. The data shown represent means + SD of independent experiments. Extreme values, also called outliers were detected with the “Median Absolute Deviation (MAD)” method, described by Leys et al. (2013). Outliers were excluded from the statistical analysis. Data were statistically evaluated with IBM SPSS Statistics for Windows, version 24.0 (IBM Corp., Armonk, NY, United States).

## Results

### SCFA Receptors Are Expressed on the Cell Surface of Multiple Cell Types of the Horse

**FIGURE 1:**
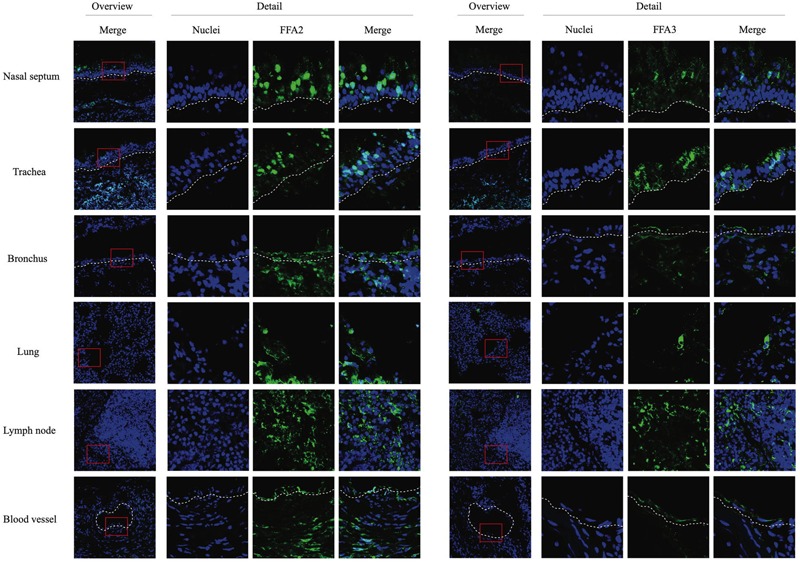
Immunofluorescence staining of the two main SCFA receptors, FFA2 (left panel) and FFA3 (right panel) in respiratory and lymph nodal tissues isolated from healthy horses. After collection, tissues were snap-frozen in liquid nitrogen. Both receptors were stained in green (FITC), nuclei were counterstained in blue (Hoechst).

Cryosections of respiratory (nasal septum, trachea, and lung) and lymph nodal tissue samples were made, followed by indirect immunofluorescence staining for the two main SCFA receptors, FFA2 and FFA3. In nasal and tracheal mucosa, both FFA2 and FFA3 receptors were diffusely expressed in the epithelium lining the luminal surface (Figure 1). FFA2 was more extensively expressed by the epithelial cells compared to FFA3. In addition, a similar expression pattern of FFA2 and FFA3 was detected in the epithelium lining the bronchial and lung lumen. Both receptors were expressed by immune cells present in the lymph nodes and by the endothelium lining the blood vessels. Taken together, SCFA receptors FFA2 and FFA3 are present at the luminal surface of the equine respiratory tract and blood vessels, and on the cell surface of circulating immune cells.

### SPr and SB, but Not SAc Hinders Primary EHV1 Replication in the Upper Respiratory Tract

**FIGURE 2:**
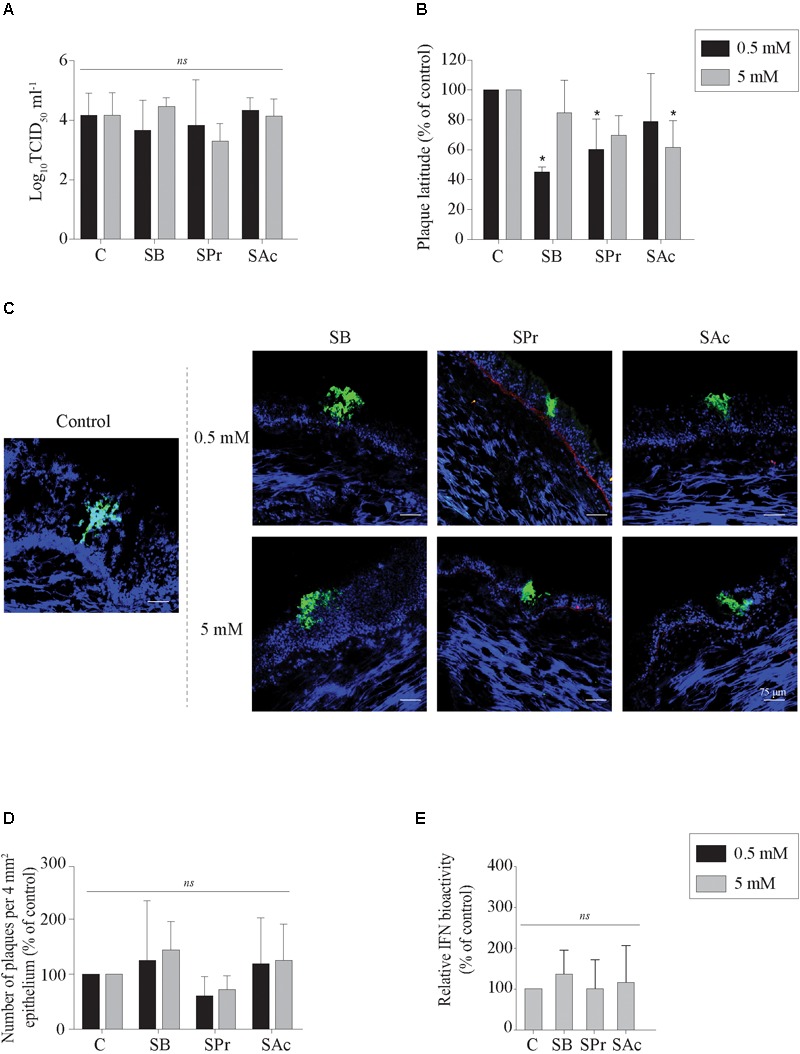
Evaluation of EHV1 replication in the presence of SB, SPr, or SAc in the upper respiratory tract. **(A)** Extracellular virus titer, and **(B)** viral plaque latitudes were analyzed at 24 hpi. **(C)** Representative confocal images of EHV1 plaques in tracheal mucosa explants are stained in green (FITC). Basement membrane is shown in red (TR), and nuclei were counterstained in blue (Hoechst). The effects of SCFA on **(D)** the number of viral plaques in tracheal mucosa explants. **(E)** Evaluation of type I interferon (IFN) bioactivity in EHV1-inoculated tracheal mucosa explants in the presence of SB, SPr, or SAc. Ten *ad random* chosen viral plaques were analyzed. Three independent experiments were performed and data are represented as means + SD. Statistical significant differences are shown with asterisks: ^∗^ *p* < 0.05; *ns* indicates *p* > 0.05.

Previous studies demonstrated that SCFA, produced by microbiota in the gastro-intestinal tract, may be absorbed through the colonic epithelium, disseminate in peripheral blood, and may reach the respiratory tract (Cummings et al., 1987; Trompette et al., 2014). Since SCFA receptors are expressed by epithelial cells in the URT, we determined whether sodium butyrate (SB), sodium propionate (SPr), and/or sodium acetate (SAc) increase primary viral replication in the URT. No significant difference in extracellular virus titer was measured between mock- or SCFA-treated mucosal explants, independent of the concentration used (Figure 2A). Viral plaques became significantly smaller compared to control (101 ± 38 μm) when the explants were pretreated with 0.5 mM SB (43 ± 4 μm) (*p* < 0.05) and 0.5 mM SPr (66 ± 46 μm) (*p* < 0.05). No significant reduction in viral plaque latitudes was detected when increasing the concentration of SB (*p* > 0.05) or SPr (*p* = 0.1) to 5 mM. Only 5 mM of SAc significantly reduced the plaque latitudes to 45 ± 19 μm (*p* < 0.05) (Figure 2B). Representative confocal images are shown in Figure 2C. The number of viral plaques in the respiratory mucosa explants pretreated with SCFA did not significantly alter compared to control (Figure 2D). Noteworthy, viral plaques in SB-treated mucosa explants were observed in the apical part of the respiratory epithelium, suggesting a more efficient plaque sequestration and viral clearance in the URT in the presence of sufficient concentrations of butyrate. Taken together, we can conclude that 0.5 mM of SB and SPr reduces the lateral spread of EHV1 in the URT, resulting in smaller viral plaques. Higher concentrations of SPr and SAc (marginally) reduced the size of the viral plaques.

### SCFA Does Not Activate the Innate Response in the Upper Respiratory Tract After EHV1 Infection

The innate recognition of alphaherpesviruses is principally orchestrated by type I interferon (IFN). In a previous study we demonstrated that type I IFN is induced upon EHV1 replication in the URT (Poelaert et al., 2018). Nusinzon and Horvath (2003) indicated a putative stimulatory role of butyrate on the phosphorylation of STAT-1 and -2, one of the first steps in the IFN-signaling pathway. Based on those reports, we hypothesized that SCFA might regulate the innate immune response in the URT of the horse, by activating the IFN signaling pathway. Therefore, the production of bioactive type I IFN was measured in the supernatants of EHV1-inoculated tracheal mucosa explants treated with 0 or 5 mM of SB, SPr or SAc. Results are shown in Figure 2E as percentage of control. No significant differences in type I IFN levels were detected between SB, SPr, or SAc treated explants (*p* > 0.05). Taken together, SCFA pretreatment did not activate the production of type I IFN in EHV1-infected respiratory mucosa explants of the horse.

### SCFA Does Not Change Infection of Blood Mononuclear Cells

Short-chain fatty acids have been demonstrated to modulate the phenotype, metabolism and function of cells of the immune system. Indeed, butyrate, propionate and acetate mediate T-lymphocyte proliferation *in vitro* (Franklin et al., 1991; Wajner et al., 1999). However, little information is available of the role of SCFA on EHV1 infections of mononuclear cells. Since EHV1 has a prominent ability to infect immune cells and more efficiently infects (IL-2) activated T-lymphocytes (van der Meulen et al., 2000; Poelaert et al., 2019), we hypothesized that SCFA might reduce EHV1 infection of mononuclear cells by interfering with immune cell proliferation or by changing cellular phenotypes, and thus impeding the cell-associated viremia.

**FIGURE 3:**
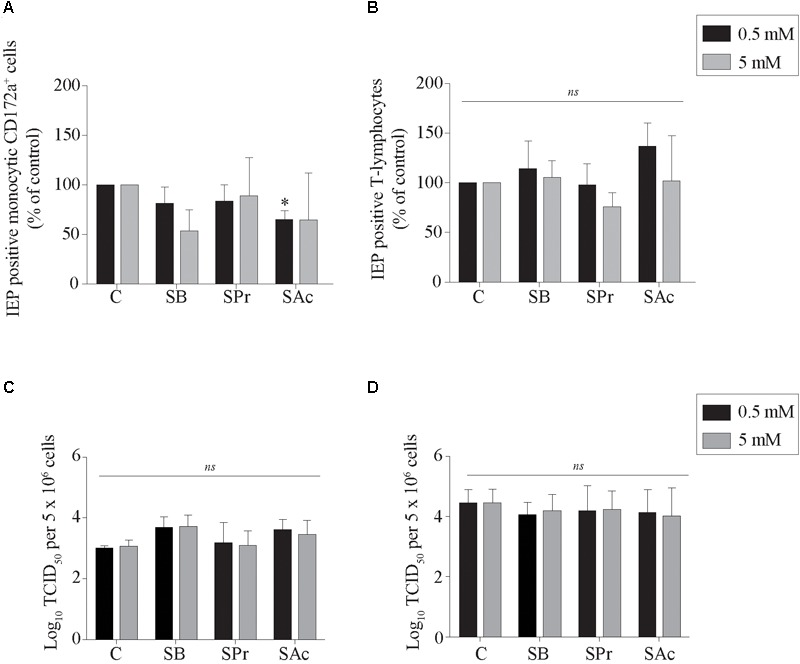
Analysis of SCFA pretreatment on EHV1 infection of mononuclear cells. The number of immediate early protein (IEP) positive **(A)** monocytic CD172a^+^ cells and **(B)** T-lymphocytes at 12 hpi. The extracellular virus titer were analyzed in the supernatants of the EHV1-inoculated **(C)** monocytic CD172a^+^ cells and **(D)** T-lymphocytes. Five *ad random* chosen fields of 100 cells were analyzed. Three independent experiments were performed and data are represented as means + SD. Statistical significant differences are shown with asterisks: ^∗^ *p* < 0.05; *ns* indicates *p* > 0.05.

Here, viral infection of the mononuclear cells in the presence or absence of SCFA was analyzed by indirect immunofluorescence staining. The number of EHV1-infected monocytic and T-cells are shown as percentage of control in Figure 3A,B. Pretreatment of monocytic cells and T-lymphocytes cells with 0.5 or 5 mM SB did not significantly alter the number of EHV1 infected cells, compared to control (*p* > 0.05). Similarly, for the cells treated and incubated with SPr showed no significant differences with regard to number of EHV1 positive cells, compared to control (*p* > 0.05). A 1.5-fold reduction in EHV1-positive monocytic CD172a^+^ cells, but not EHV1-positive T-cells, was observed upon pretreatment and cultivation with 0.5 mM SAc (*p* < 0.05). However, increasing the concentration of SAc did not significantly alter the number of EHV1 infected monocytic cells (*p* > 0.05).

Supernatants of EHV1-inoculated immune cells were analyzed by virus titration to quantify the extracellular virus titers. A marginally higher virus titer was observed when the monocytic cells were pretreated with 0.5 and 5 mM SB, compared to control (*p* = 0.179 and *p* = 0.165, respectively) (Figure 3C,D). In contrast, no changes in virus titer were detected when monocytic CD172a^+^ cells were treated with SPr (*p* = 0.909 and *p* = 0.999, respectively), or SAc (*p* = 0.237 and *p* = 0.497, respectively). Treatment of the T-lymphocytes with SFCA did not affect the extracellular virus titer. Taken together, these results indicate that SCFA do not directly affect the ability of EHV1 to infect monocytic CD172a^+^ cells or T-lymphocytes. Only SAc reduced the number of EHV1 infected monocytic cells at the concentration of 0.5 mM. EHV1 replication in CD172a^+^ mononuclear cells was slightly more productive in the presence of SB.

### SCFA Reduces the Adhesion of Blood-Derived Monocytic Cells and T-Lymphocytes to Equine Endothelial Cells

**FIGURE 4:**
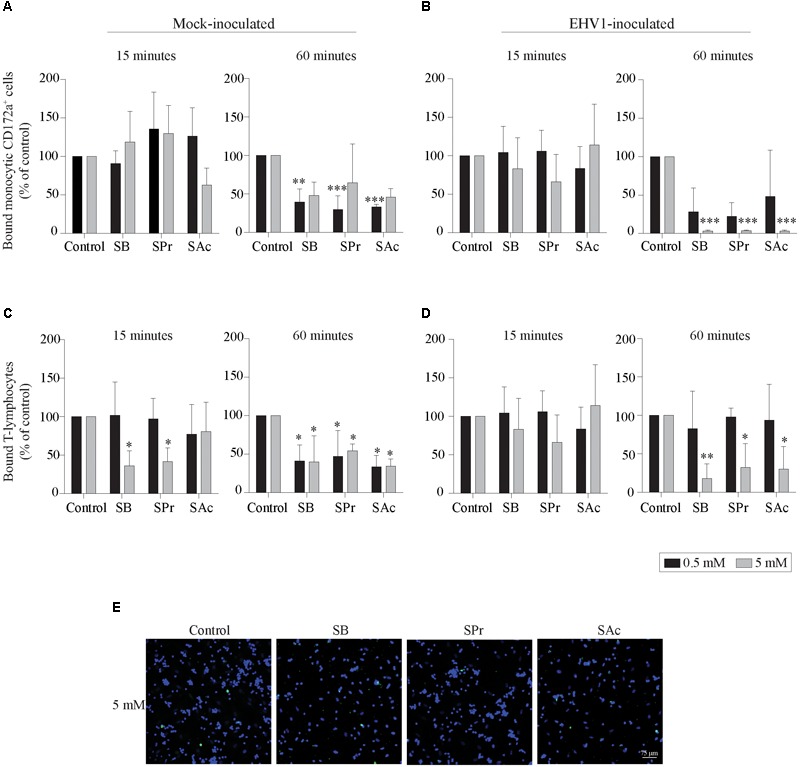
Short-chain fatty acid reduces the adhesion of mock- or EHV1-inoculated mononuclear cells to the target endothelium. **(A,B)** Monocytic cells or **(C,D)** T-lymphocytes were co-cultured during 15 (left panel) or 60 min (right panel) with 0, 0.5 or 5 mM SB, SPr, or SAc equine EC. Adherent mononuclear cells were counted per 100 EC in 5 *ad random* chosen fields. Viral proteins were stained in green (FITC). **(E)** Representative confocal images of the binding of EHV1-inoculated mononuclear cells to the target endothelium, pretreated with SCFA. Three independent experiments were performed and data are represented as means + SD. Statistical significant differences are shown with asterisks: ^∗^ *p* < 0.05; ^∗∗^ *p* < 0.01; ^∗∗∗^ *p* < 0.001; *ns* indicates *p* > 0.05.

Since the adhesion of EHV1-infected mononuclear cells is a crucial step in viral transfer to the target endothelium, we analyzed the effects of SCFA on the equine endothelial cells (EC). Mock- or EHV1-inoculated monocytic cells and T-lymphocytes were used as model to analyze the binding capacities of PBMC to SCFA-treated target EC. After 15 min of co-cultivation with 0.5 mM SCFA, no significant differences in binding capacity of mock- or EHV1-inoculated monocytic cells or T-lymphocytes were observed between control and the different SCFA pretreatments. Upon pretreatment with 5 mM SB and SPr, the percentage of bound mock-inoculated T-cells decreased to 36 ± 19% (*p* < 0.05) and 41 ± 18% (*p* < 0.05), respectively, compared to 100% bound T-cells in control-treated EC (Figure 4A–D, left panels). This trend of reduction increased upon 60 min of co-culture. Indeed, pretreatment with 0.5 mM significantly reduced the number of bound mock-inoculated monocytic and T-cells to the EC surface (Figure 4A,C, right panel). Increasing the concentration of all SCFA did not further reduce the number of adherent mononuclear cells to the EC surface, compared to control. Upon 60 min co-cultivation of EHV1-inoculated monocytes and 0.5 mM SB or SPr pretreated EC, the percentage of bound monocytic cells decreased to 28 ± 31% (*p* = 0.086) or 22 ± 18% (*p* = 0.063), compared to 100% bound monocytes in the control conditions. Pretreatment of EC with 0.5 mM SAc did not alter the percentage of bound monocytic cells to the EC (48 ± 60%) (*p* = 0.242). Similar results were observed for T-lymphocytes upon pretreatment with 0.5 mM SB, SPr, or SAc. Treatment with 5 mM SB or SPr resulted in a 30-fold (*p* < 0.01) reduction of bound monocytic cells, compared to control. Similarly, a 5-fold (*p* < 0.05) or 3-fold (*p* < 0.05) reduction of bound T-lymphocytes was detected upon treatment with 5 mM SB, or SPr, respectively. Comparably, pretreatment with 5 mM SAc reduced the number of adherent monocytes (*p* < 0.01) and T-lymphocytes (*p* < 0.05) (Figure 4B,C, right panels). Figure 4E shows representative confocal images of the binding of EHV1-inoculated mononuclear cells to the target endothelium, pretreated with SCFA. These results show that SCFA treatment reduces the number of adherent immune cells to the endothelium.

**FIGURE 5:**
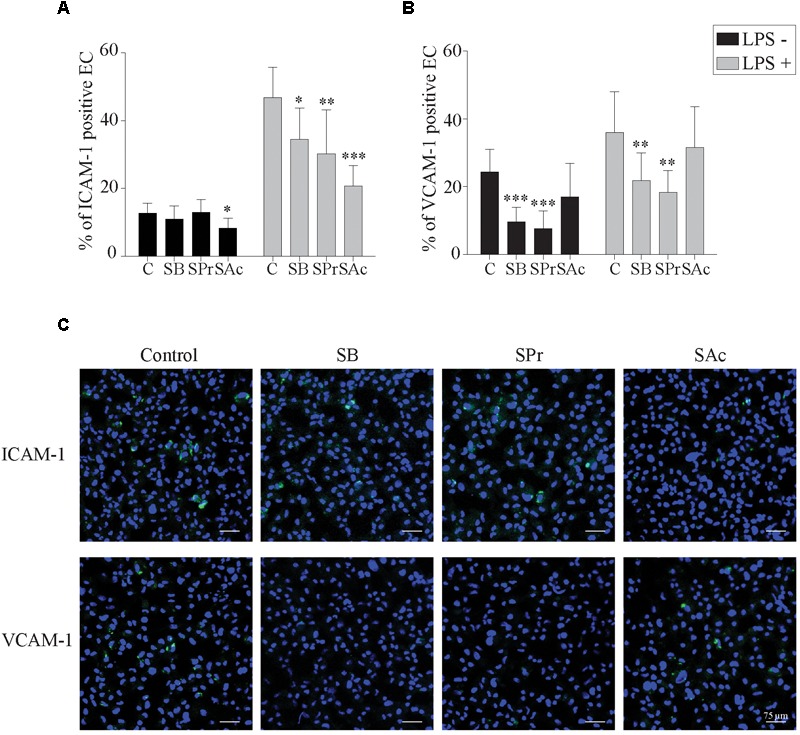
Short-chain fatty acid reduces the expression of adhesion molecules on the endothelial cell surface. The expression of the adhesion molecules **(A)** ICAM-1 and **(B)** VCAM-1 on the EC cell surface after 24 h SCFA treatment, in the presence or absence of LPS, was analyzed. **(C)** Representative confocal images of the expression of ICAM-1 and VCAM-1 in the presence or absence of different SCFA. ICAM-1, and VCAM-1 were stained in green (FITC), and nuclei were counterstained in blue (Hoechst 33342). Three independent experiments were performed and data are represented as means + SD. Statistical significant differences are shown with asterisks: ^∗^ *p* < 0.05; ^∗∗^ *p* < 0.01; ^∗∗∗^ *p* < 0.001.

To better understand this reduced adhesion, we analyzed whether the expression of ICAM-1 and VCAM-1 adhesion molecules on the endothelial cell surface was downregulated after SCFA treatment. To this end, we incubated endothelial cells with SB, SPr, or SAc, followed by the pretreatment with or without LPS, during 3 h. Since LPS stimulates the upregulation of ICAM-1 and VCAM-2 (Faure et al., 2000; Hijiya et al., 2002; Zeuke et al., 2002; Dauphinee and Karsan, 2006), we analyzed whether SCFA interfered with the LPS-mediated upregulation of those adhesion molecules. In the absence of LPS, only 13 ± 3% of the EC expressed ICAM-1 on their plasma membrane (Figure 5A). Pretreatment with SB, or SPr, did not substantially affect this percentage (11 ± 4%, 13 ± 4%, respectively). Pretreatment with SAc decreased the ICAM-1 expression on the endothelial surface (8 ± 3%) (*p* < 0.05). Treatment with LPS resulted in a significant upregulation of ICAM-1 on the cell surface (*p* < 0.001). SB (*p* < 0.05), SPr (*p* < 0.01) and SAc (*p* < 0.001) pretreatment significantly reduced the ICAM-1 expression on the EC surface, compared to control.

VCAM-1 expression on EC without LPS-treatment, showed marginal differences between SAc (17 ± 10%) and control (24 ± 7%) treated EC (*p* = 0.062) (Figure 5B). Treatment with SB (10 ± 4%) or SPr (8 ± 5%) did downregulate VCAM-1 expression on the plasma membrane, *p* < 0.001, and *p* < 0.001, respectively. When EC were treated with LPS, a significant upregulation of VCAM-1 was observed (*p* < 0.001), though a similar reduction in the VCAM-1 expression was observed when treated with SB (22 ± 8%) (*p* < 0.01), SPr (18 ± 6%) (*p* < 0.01), compared to control (36 ± 12%). No differences were observed between control-treated and SAc-treated EC (32 ± 12%) (*p* = 0.641). Representative confocal images are shown in Figure 5C. Taken together, it can be concluded that SCFA efficiently downregulate ICAM-1 and VCAM-1 on the plasma membrane of EC.

### SCFA Indirectly Inhibits the Viral Transfer From Mononuclear Cells to Equine Endothelial Cells

**FIGURE 6:**
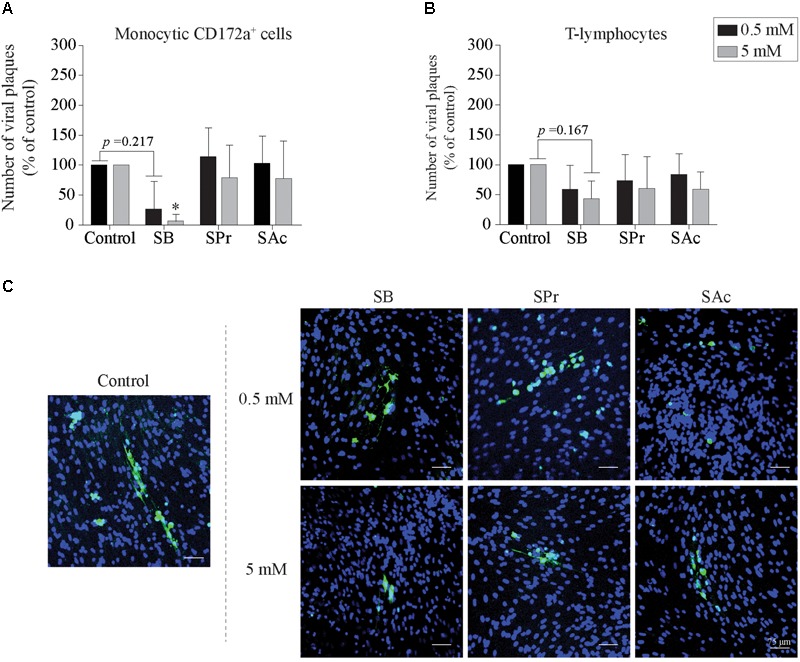
Viral transfer from mononuclear cells to SCFA-pretreated endothelial cells (EC). The number of viral plaques in SCFA-treated EC were analyzed after 36 h of co-cultivation with EHV1-inoculated **(A)** monocytic CD172a^+^ cells, or **(B)** T-lymphocytes. Number of plaques were counted per 0.25 cm^2^. Three independent experiments were performed and data are represented as means + SD. Statistical significant differences are shown with asterisks: ^∗^ *p* < 0.05. **(C)** Representative confocal images of EHV1 viral plaques in EC. Viral proteins were shown in green (FITC), nuclei were counterstained in blue (Hoechst).

Next, we checked whether SCFA-mediated downregulation of ICAM-1 and VCAM-1 eventually results in reduced viral transfer from EHV1-inoculated monocytic CD172a^+^ cells and T-lymphocytes to EC. To this end, the number of viral plaques upon 36 h of co-cultivation with EC was determined, shown in Figure 6. The number of viral plaques upon co-cultivation of monocytic cells and EC was comparable between untreated and SPr-, and SAc-treated EC (Figure 6A). However, when the EC were pretreated with 0.5 or 5 mM SB prior to the co-cultivation with EHV1-inoculated monocytic cells, a reduction in the number of viral plaques was observed. Only 7 ± 11 viral plaques were detected in the EC treated with 5 mM SB, compared to the 100 viral plaques in the control (*p* < 0.05). Similar results were observed when EHV1-inoculated T-lymphocytes were co-cultured with SCFA-treated EC (Figure 6B). The number of viral plaques significantly reduced from 100 in the control-treated EC, to 43 ± 30 plaques when treated with 5 mM SB (*p* = 0.167). Representative confocal images are shown in Figure 6C.

In conclusion, upon pretreatment of EC with 0.5 and 5 mM SB, we demonstrated a reduced number of viral plaques upon co-cultivation of EHV1-inoculated mononuclear cells with EC. In contrast, SPr and SAc did not change the number of viral plaques in the target endothelium.

### SCFA Does Not Inhibit Viral Replication in Equine Endothelial Cells

**FIGURE 7:**
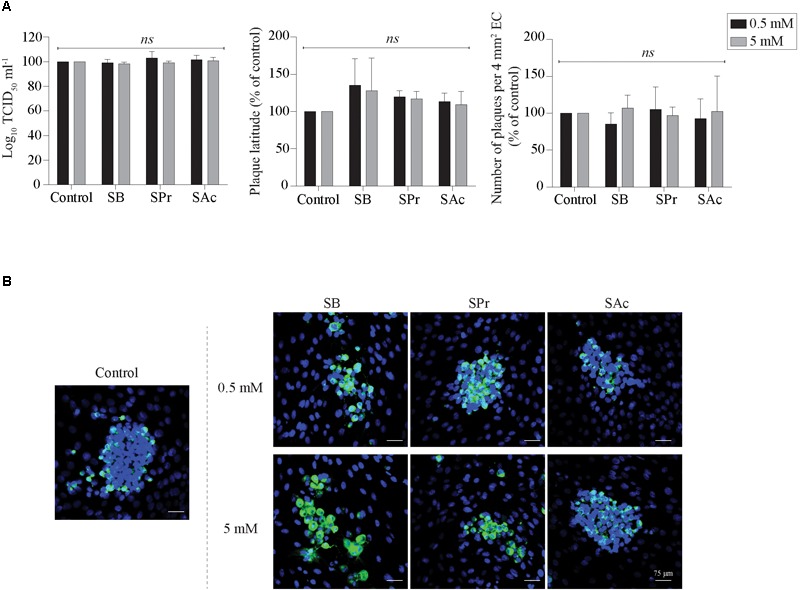
Analysis of SCFA pretreatment on EHV1 infection of equine EC. **(A)** Extracellular virus titer (left), viral plaque latitudes (middle), and number of plaques (right) were analyzed at 24 hpi. **(B)** Representative confocal images of EHV1 plaques in EC are stained in green (FITC). Nuclei were counterstained in blue (Hoechst). Five *ad random* chosen viral plaques were analyzed. Three independent experiments were performed and data are represented as means + SD. Statistical analysis was performed: *ns* indicates *p* > 0.05.

Since Li et al. (2018) demonstrated the anti-inflammatory effects of SCFA on endothelial cells, we analyzed whether SCFA directly affects EHV1 replication in EC. Therefore, the extracellular virus titer, number of plaques and plaque latitude were determined in EHV1-inoculated EC upon treatment with 0.5 or 5 mM SB, SPr, or SAc. No significant alterations in replication kinetics of EHV1 was observed upon pretreatment, inoculation, and cultivation in the presence of 0.5 or 5 mM SB, SPr, or SAc (Figure 7A). Representative confocal images are shown in Figure 7B. Altogether, we can conclude that SCFA do not have a significant effect on the viral replication in equine EC *in vitro*.

## Discussion

The mammalian gut represents a complex ecosystem consisting of an extraordinary number of resident commensal bacteria which are involved in the development and function of the immune response (Chow et al., 2010). In addition, bacterial fermentation metabolites of carbohydrates, such as SCFA, are known for their protective effects on epithelial cells in the gut. Once absorbed in the systemic circulation, they positively affect the general host physiology, including cardiovascular function, inflammation, and atherosclerosis (Esposito and Giugliano, 2006; Anderson et al., 2009; Morrison and Preston, 2016). Interestingly, Trompette et al. (2014) demonstrated that dietary fibers shape the composition of the intestinal, and to a lesser extent the airway microbiota in mice. This resulted in major differences in allergic airway responses in mice. These results are in line with epidemiological studies, showing that dietary fibers reduce the incidence of asthma in humans, associated with a sophisticated cross-talk between the gastrointestinal and respiratory tract (Marsland et al., 2015).

Horses are extremely susceptible to disturbance of their gut microbiota, often resulting in gastro-intestinal disorders. Because of the increased demands placed on horses for athletic performances, the fiber-based diet is often supplemented with varying quantities of high energetic grains and oils (Dougal et al., 2014). An overload of cereal starch might result in the rapid microbial fermentation in the hindgut, resulting in increased lactic acid. This can result in a decreased luminal pH, which suppresses the growth of obligate fibrolytic acid-intolerant bacteria, leading to a reduced production of SCFA.

Based on the proven importance of SCFA on the overall physiology of mammals and the delicate balance of the microbiome of horses, we hypothesized that systemic fluctuations of SCFA levels might influence the pathogenesis of viral infections, which might contribute to individual differences in clinical outcome. Since EHV1 is a major equine pathogen, which horses almost invariably encounter at some point in life, we examined the putative protective effects of SCFA upon primary EHV1 replication in the URT, viremia, viral transfer to and replication in the target endothelium.

First we focused on the primary site of EHV1 replication, the URT. We demonstrated that the two main SCFA receptors, FFA2 and FFA3, are overall expressed in the respiratory tract, at the cell surface of immune cells, and endothelium lining the blood vessels of the horse. These results are in line with the study of Imoto et al. (2018), demonstrating the prevalence of both receptors in human sino-nasal tissues. The interaction of SCFA and FFA2/FFA3 modulates and controls inflammatory responses, including airway diseases in humans (Trompette et al., 2014). Despite the described protective effects of SCFA in respiratory disease, we were not able to detect major differences in primary EHV1 replication in the URT, after SCFA pretreatment. Virus titer, plaque latitudes or number of plaques were not altered in the presence of SCFA. Only SB and SPr reduced the EHV1 plaque latitudes. This could be due to the expression of FFA2 and FFA3, and the efficiency for SCFA binding. Indeed, at epithelial cell surfaces, SPr and SB bind more efficiently to FFA2 and/or FFA3, while this binding is less effective for SAc (Li et al., 2018). In addition, we found an enhanced plaque sequestration in the presence of SB. One speculative explanation may be the enhanced mitosis of basal cells in the respiratory epithelium in the presence of SB (Reichert et al., 2012). However, immunofluorescence staining of the G2/M-phase specific protein cyclin B1 could not confirm this hypothesis (data not shown). In addition, butyrate is the most potent HDAC inhibitor of all three tested SCFA (Schilderink et al., 2013), facilitating efficient binding of transcription factors to cellular chromatin, suggesting an enhanced cell cycle progression. Another putative explanation could be found in a modulated type I IFN response, leading to the plaque sequestration. Nusinzon and Horvath (2003) described a butyrate-mediated enhancement of the phosphorylation of STAT-1 in the IFN signaling pathway. However, our data are not in line with such hypothesis, since no increase or decrease in IFN bioactivity could be observed in the presence of SB, SPr, or SAc. More research is required to explain the mechanism of plaque sequestration in the presence of SB.

During EHV1 infection, extravasated monocytic CD172a^+^ cells and T-lymphocytes are attracted to the infected respiratory mucosa within 24–36 hpi. Infecting the cellular arm of the immune response may represent an effective immune evasion strategy of EHV1 to evade the immune response, to promote viral dissemination in the host, evade host clearance and establish latency (van der Meulen et al., 2000; Gryspeerdt et al., 2010; Vandekerckhove et al., 2010; Laval et al., 2015a, 2017). Here, we demonstrated that both SCFA receptors were expressed on the cell surface of leukocytes in the draining lymph nodes, suggesting that SCFA may affect leukocytes. It has been reported that SCFA could enter immune cells in a FFA2/FFA3 dependent or independent manner, via passive diffusion (Smith et al., 2013). Once entered in leukocytes, SCFA modulates the systemic inflammation and infection by mediating HDAC activity (Ang and Ding, 2016). Interestingly, Laval et al. (2015a) demonstrated that the replication of abortigenic EHV1 variants in monocytic CD172a^+^ cells is silenced and strictly regulated by HDAC. Treatment of the monocytic CD172a^+^ cells with butyrate resulted in activation of viral replication, with transcription and translation of the late viral proteins. In addition, several studies indicated that SCFA shape the T-cell response, by activating the mTOR signaling pathway, and by regulating the differentiation of CD4^+^ T-cells into Th1 and Th17 cells (Park et al., 2015). Since SCFA changes the leukocyte phenotype, we focused on the two main target cells of EHV1 viremia, monocytic CD172a^+^ cells and T-lymphocytes. Pretreatment of these immune cells with different concentrations of SB, SPr, or SAc did not notably increase the number of EHV1 positive cells. This indicates that SCFA does not change the susceptibility of mononuclear cells to EHV1 infection. However, we demonstrated that extracellular virus titers slightly increased when monocytic cells were treated with SB, but not with SPr or SAc. This is not surprising, since principally SB, and to a lesser extent SPr, are effective HDAC inhibitors (Schilderink et al., 2013). Treatment of T-lymphocytes with SCFA did not alter the extracellular virus titer, suggesting that HDAC may not be involved in EHV1 replication or egress in T-cells.

Following the cell-associated viremia, EHV1-infected immune cells reach the endothelium lining the blood vessels of the pregnant uterus or CNS. During this stage of infection adhesion molecules expressed on both immune cells and EC play a pivotal role in EHV1 transfer to the endothelium (Laval et al., 2015b). The firm adhesion of leukocytes to vascular endothelium is mediated through ICAM-1 and VCAM-1. Here, we showed that pretreatment of EC with SB, SPr, or SAc inhibited the expression of VCAM-1, and to a lesser extent ICAM-1 on the EC surface. ICAM-1 serves as an endothelial counter-receptor for several leukocyte surface molecules, such as α_L_β_2_ integrin (or LFA1), and VCAM-1 is the endothelial counter-receptor for α_4_β_1_ integrin (or VLA-4) (Springer, 1994). Interestingly, LFA1 and VLA4 play a fundamental role in the contact between an EHV1-infected monocytic CD172a^+^ cells and the adjacent EC (Laval et al., 2015b). This interaction facilitates direct viral transfer from the infected monocytic cell, to the engaged endothelium. Thus, SCFA-mediated downregulation of the endothelial cell adhesion molecules may lead to reduced adhesion of mononuclear cells to EC, and subsequently reduced viral transfer and infection of EC. Our results are in line with Menzel et al. (2004) and Zapolska-Downar et al. (2004), indicating that butyrate downregulates the NFκB signaling pathway, resulting in the downregulation of ICAM-1 and VCAM-1 in human umbilical endothelial cells (HUVEC). However, we are the first to demonstrate that SCFA may have a protective effect at the level of the target endothelium during systemic EHV1 infection. We believe that these results can be extended to other mammalian (herpes)viruses. Several common human viruses, including herpes simplex virus 1, human cytomegalovirus, pseudorabies virus and measles virus, transfer infection from infected mononuclear cells, to target endothelium (Friedman et al., 1981; Gosselin et al., 1992; Van de Walle et al., 2003; Goodrum and Bughio, 2015). More research is required to unravel the beneficial effects of SCFA in the pathogenesis of other life-threatening viruses.

In conclusion, our results indicate that physiological SCFA levels temper the EHV1 pathogenesis *ex vivo* and *in vitro*. SB and SPr mainly inhibits the lateral spread of EHV1 in the URT, while SAc reduces the number of EHV1-infected monocytic CD172a^+^ cells. SB decreases the adhesion of infected mononuclear cells to EC by reducing the membrane expression of crucial endothelial adhesion molecules. These results contribute to the hypothesis that clinical EHV1 manifestations can be diminished by correct management and nutrition.

## Ethics Statement

This study was reviewed and approved by the Ethical Committee of Ghent University. The protocols were approved by the Ethical Committee.

## Author Contributions

KP designed and performed all the experiments, statistically evaluated the results, designed the figures, and wrote the first draft of the manuscript. JVC and SD helped to perform experiments. KL helped to design the experiments. HF and HN were the promoters of KCP, and designed the experiments. All authors reviewed the manuscript.

## Conflict of Interest Statement

The authors declare that the research was conducted in the absence of any commercial or financial relationships that could be construed as a potential conflict of interest.
